# Supplementary material for: Impact of rising temperatures on the bacterial communities of Aphaenogaster ants
Source: Biol Open. 2025 Aug 15;14(8):bio062145. doi: 10.1242/bio.062145 (PMC12381927; doi:10.1242/bio.062145)
Supplement: Supplementary information [file biolopen-14-062145-s1.pdf]

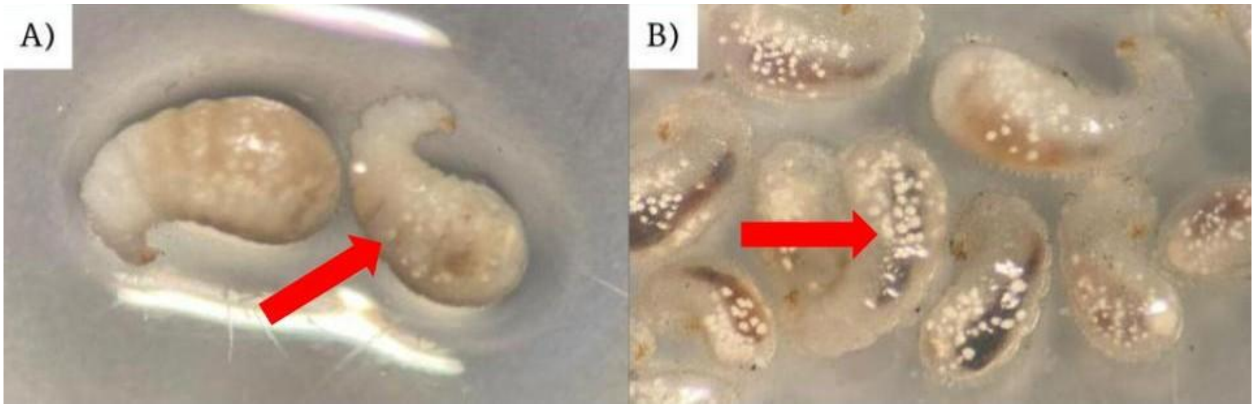

Fig. S1. Comparison of larvae from the Control (**A**) and the Experimental group (**B**). This image highlights the differences in the fat storges observed in the control and experimental groups. Larvae themselves are cream colored, and comma shaped. The fat storages are the small white dots on the larvae that are indicated by the red arrows.

Biology Open • Supplementary information

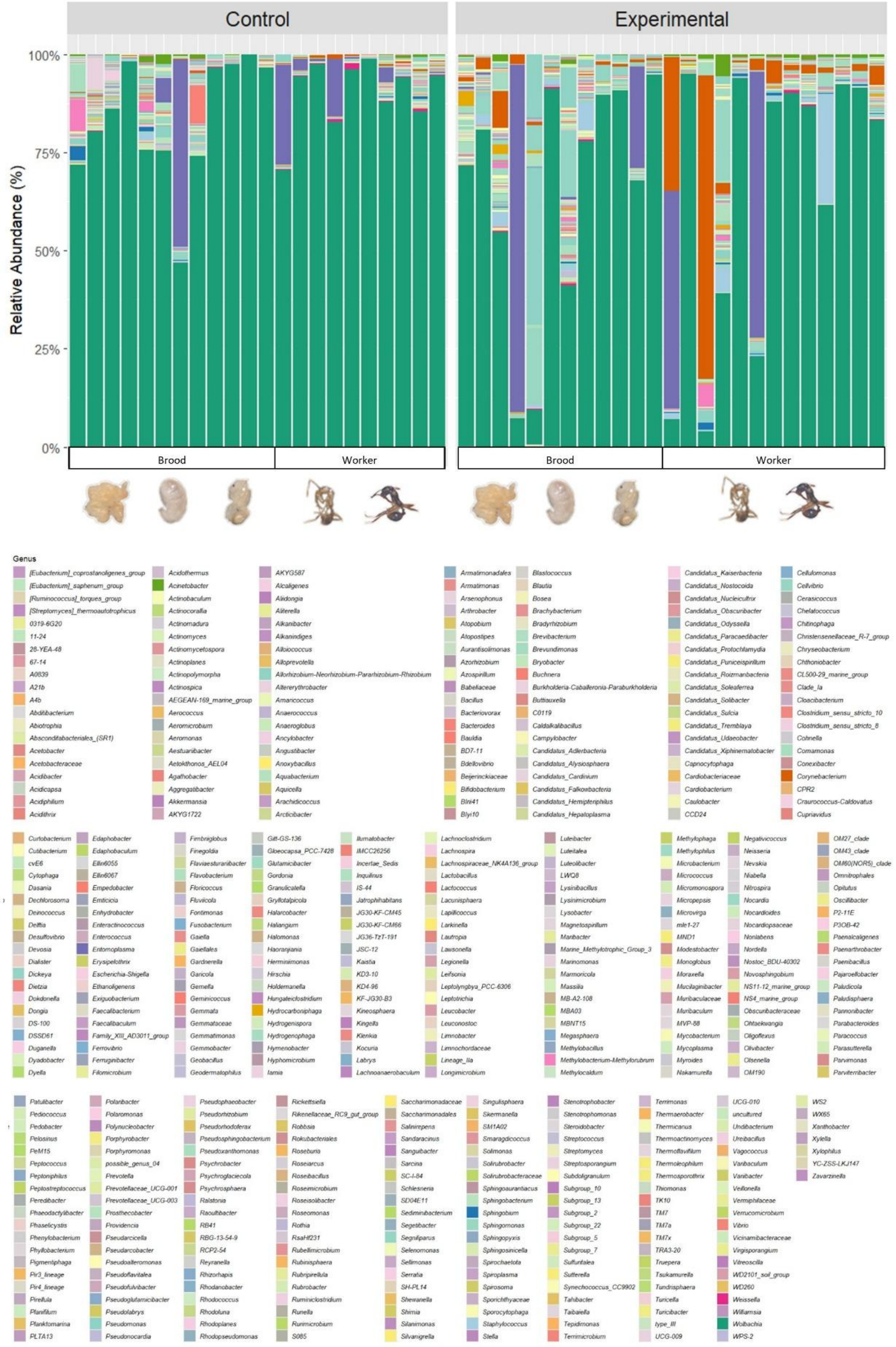
Control
Experimental
Relative Abundance (%)
100%
75%
50%
25%
0%
Brood
Worker
Brood
Worker
Genus

Fig. S2. Stacked bar cart of the whole bacterial community (ASVs) at the genus level found in *Aphaenogaster rudis*. Each column represents a sample, and each color represents a bacteria genus (ASV). There was found to be a total of 7,951 bacterial ASVs in the bacterial communities of the *Aphaenogaster* samples.

Biology Open • Supplementary information

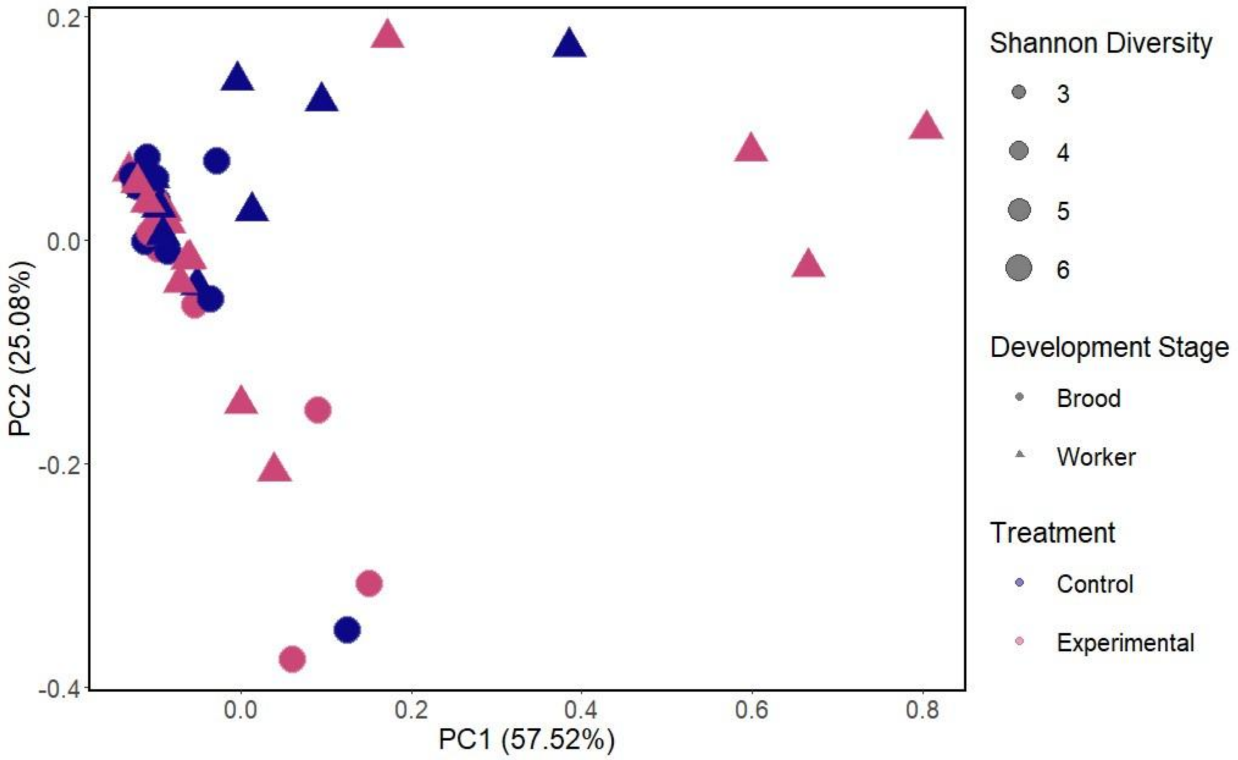

Fig. S3. PCoA plots showing weighted unifrac distance matrix. Size is based on alpha diversity, the larger the dots the higher the diversity in the sample (Kruskal-Wallis-Shannon Diversity; $H = 4.025$; *p-value* = 0.403) The closer the dots are to one another the more similar the composition and abundance of the bacterial communities. Dots are colored by treatment and shaped as development stage.

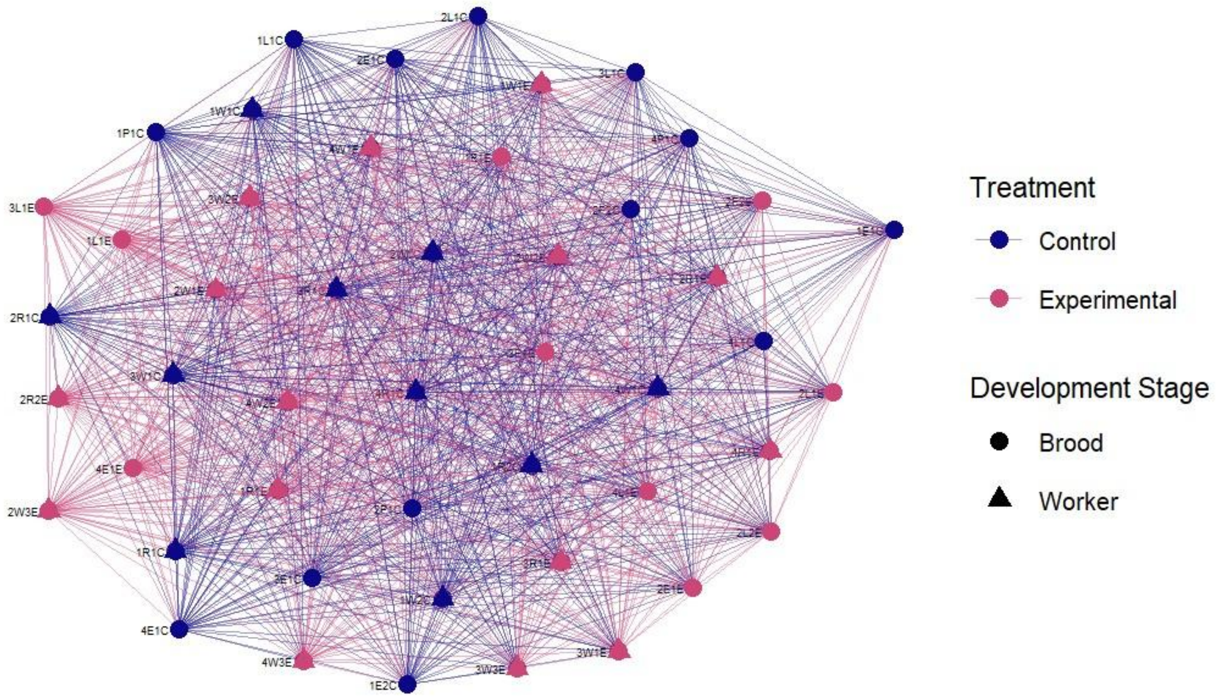

Fig. S4. Network maps showing unweighted unifrac distance matrix. Each dot represents a sample, identified by their sample ID and the closer the dots are to each other the more similar the bacterial communities. The lines indicate the interactions occurring between the samples. Dots are colored by treatment and shaped as a development stage.

Biology Open • Supplementary information

Table S1. Identification of *Aphaenogaster rudis* colonies used in the study. The species was determined by using BLAST with the COI gene and selecting the identification with the highest similarity percentage.

Colony	Ant Species	Query Cover (%)	E Value	Percent Similarity (%)	Accession Code from NCBI for Top Hit	Accession Code for Study Sample in NCBI
A	*A. rudis*	96.0	0.0	100.00	KP730107.1	SRR30552027
B	*A. rudis*	97.0	0.0	100.00	KP730107.1	SRX28262940
C	*A. rudis*	93	0.0	99.53	KJ920540.1	SRX28262942
D	*A. rudis*	98.0	0.0	99.84	KP730107.1	SRR30552035

Table S2. Mortality data collected from the *Aphaenogaster* colonies. Colony A-D were subjected to changing temperatures and the Control colony was kept at 22 °C. The values in the column for each colony represents the number of dead ants collected on that day. Colony A had a total population of 114 ants, Colony B had a total population of 120 ants, Colony C had a total population of 47 ants, Colony D had a total population of 303 ants, and the Control had a total population of 350 ants.

Day	Temperature	Colony A	Colony B	Colony C	Colony D	Control
1	23	5	2	1	3	1
2	23	2	4	1	1	2
3	24	2	2	2	1	3
4	24	2	1	1	0	0
5	24	0	1	0	5	2
6	25	0	1	0	3	3
7	25	0	5	1	2	2
8	25	2	0	0	0	1
9	26	4	1	0	2	2

10	26	1	0	1	0	0
11	26	0	1	0	0	1
12	27	1	2	0	0	3
13	27	0	2	0	1	2
14	27	4	1	0	3	1
15	28	0	0	0	0	4
16	28	2	0	3	0	3
17	28	0	1	1	4	2
18	29	0	0	0	0	1
19	29	2	0	0	4	2
20	29	1	3	0	3	3

Biology Open • Supplementary information

21	30	3	0	0	4	2
22	30	2	1	0	1	2
23	30	1	2	0	0	1
24	31	4	3	2	2	1
25	31	0	1	2	0	0
26	31	3	0	1	5	0
27	32	1	3	1	4	1
28	32	1	0	2	0	2
29	32	2	3	0	4	3
30	32	1	1	0	3	0
31	32	3	2	0	5	2

Biology Open • Supplementary information

32	32	29	3	3	37	3
33	32	3	7	2	6	1
34	32	4	5	0	16	2
35	32	4	8	0	20	2
36	32	3	8	3	20	3
37	32	5	3	3	19	3
38	32	4	7	4	34	1
39	32	0	1	1	1	0
40	32	1	8	0	13	1
41	32	2	2	0	35	1
42	32	5	5	8	32	2

Biology Open • Supplementary information

Table S3. Abundance (permanova weighted unifrac distance) and comparison (permanova unweighted unifrac distance) analysis of microbiota associated with *Aphaenogaster* rudis based on development stage and post hoc pairwise. Permanova analysis of bacterial communities present in different development stages. The values highlighted in red are significant p-values.

Comparison	p-value	Test statistic (pseudo-F)
Development Stage Overall	0.032	2.123
Egg vs. Larvae	0.911	0.455
Egg vs. Pupa (We recognize that we have less pupa than other development stages to present)	0.294	1.017
Egg vs. Recently Emerged Worker	0.455	0.880
Egg vs. Worker	0.062	3.170

Larvae vs. Pupa (We recognize that we have less pupa than other development stages to present)	0.352	1.011
Larvae vs. Recently Emerged Worker	0.914	0.479
Larvae vs. Worker	0.089	2.791
Pupa vs. Recently Emerged Worker (We recognize that we have less pupa than other development stages to present)	0.226	1.324
Pupa vs. Worker (We recognize that we have less pupa than other development stages to present)	0.150	1.923
Recently Emerged Worker vs. Worker	0.150	1.922

Biology Open • Supplementary information
